# Comparison of Three Nutritional Screening Tools with the New Glim Criteria for Malnutrition and Association with Sarcopenia in Hospitalized Older Patients

**DOI:** 10.3390/jcm9061898

**Published:** 2020-06-17

**Authors:** Francesco Bellanti, Aurelio Lo Buglio, Stefano Quiete, Giuseppe Pellegrino, Michał Dobrakowski, Aleksandra Kasperczyk, Sławomir Kasperczyk, Gianluigi Vendemiale

**Affiliations:** 1 Department of Medical and Surgical Sciences, University of Foggia, viale Pinto 1, 71122 Foggia, Italy; aurelio.lobuglio@unifg.it (A.L.B.); stefanoquiete@gmail.com (S.Q.); giuseppe_pellegrino.539013@unifg.it (G.P.); gianluigi.vendemiale@unifg.it (G.V.); 2 Department of Biochemistry, Faculty of Medical Sciences in Zabrze, Medical University of Silesia in Katowice, Jordana 19, 41-808 Zabrze, Poland; michal.dobrakowski@poczta.fm (M.D.); olakasp@poczta.onet.pl (A.K.); kaslav@mp.pl (S.K.)

**Keywords:** nutritional status, sarcopenia, nutritional screening tools, hospitalized older patients

## Abstract

The integrated assessment of nutritional status and presence of sarcopenia would help improve clinical outcomes of in-hospital aged patients. We compared three common nutritional screening tools with the new Global Leadership Initiative on Malnutrition (GLIM) diagnostic criteria among hospitalized older patients. To this, 152 older patients were assessed consecutively at hospital admission by the Malnutrition Universal Screening Tool (MUST), the Subjective Global Assessment (SGA), and the Nutritional Risk Screening 2002 (NRS-2002). A 46% prevalence of malnutrition was reported according to GLIM. Sensitivity was 64%, 96% and 47%, and specificity was 82%, 15% and 76% with the MUST, SGA, and NRS-2002, respectively. The concordance with GLIM criteria was 89%, 53% and 62% for the MUST, SGA, and NRS-2002, respectively. All the screening tools had a moderate value to diagnose malnutrition. Moreover, patients at high nutritional risk by MUST were more likely to present with sarcopenia than those at low risk (OR 2.5, CI 1.3-3.6). To conclude, MUST is better than SGA and NRS-2002 at detecting malnutrition in hospitalized older patients diagnosed by the new GLIM criteria. Furthermore, hospitalized older patients at high risk of malnutrition according to MUST are at high risk of presenting with sarcopenia. Nutritional status should be determined by MUST in older patients at hospital admission, followed by both GLIM and the European Working Group on Sarcopenia in Older People (EWGSOP2) assessment.

## 1. Introduction

The average population age is increasing in developed countries, causing a rise in older subjects with consequently greater need of hospitalization [1]. In such scenario, the association between hospitalization and malnutrition is increasingly reported, with a negative impact on treatment response, functional recovery, hospital length-of-stay and costs, and quality of life [2,3]. Hospitalization is also linked to loss of muscle mass and strength, which define sarcopenia [4]. Malnutrition is strongly associated with sarcopenia, and the presence of both conditions is related to several adverse outcomes [5]. The concomitant occurrence of malnutrition and sarcopenia is defined as malnutrition-sarcopenia syndrome (MSS), which represents a prognostic factor for hospitalized older adults [6]. The integrated assessment of nutritional status and presence of sarcopenia would help improve clinical outcomes of such patients.

Diagnosis of malnutrition or risk of malnutrition requires a comprehensive nutritional assessment, which is frequently difficult to perform on all in-hospital patients due to both time and financial restraints [7]. To overcome this limitation, the Global Leadership Initiative on Malnutrition (GLIM) recommends a two-step model in which diagnosis assessment is preceded by risk screening using any validated tool [8]. Nevertheless, despite several tools for rapid identification of malnutrition in older adults [9], patients are not consistently screened for nutritional status at hospital admission [10,11].

The Mini Nutritional Assessment (MNA) is considered one of the most validated tools for the identification of malnutrition or risk of malnutrition, and it is particularly used in older people [12,13,14]. However, the MNA has disadvantages such as subjective questions unsuitable to hospitalized older people, inability to be used in patients with cognitive impairment, and 10 to 15 min to be performed [7,15]. Several nutritional screening tools have been applied to rapidly identify malnutrition in older patients in hospital settings, and each present with improvements and weaknesses [7]. Very recently, a systematic review evaluated the available studies which considered malnutrition and sarcopenia simultaneously, resulting in methodological unpredictability [16].

First, this study aimed to compare different tools for nutritional screening, such as the Malnutrition Universal Screening Tool (MUST), the Subjective Global Assessment (SGA), and the Nutritional Risk Screening 2002 (NRS-2002) in hospitalized older patients, in order to define their sensitivity, specificity, and rapidity with respect to the GLIM consensus, chosen as the reference method. Furthermore, the present investigation evaluated the association between the alteration of nutritional status identified by these tools and the presence of sarcopenia.

## 2. Experimental Section

### 2.1. Study Design and Participants

We collected and analyzed data from older patients hospitalized at the Internal and Aging Medicine clinic of the “Ospedali Riuniti”, a teaching hospital in Foggia (Italy). We recruited consecutive patients aged 65 years or older, admitted to our ward from March 2019 to February 2020. Frequency of the main causes for hospital admission is reported in Supplementary Table S1. The exclusion criteria were the following: dysphagia, active cancer, severe cognitive impairment (assessed with a Mini Mental State Examination score ≤ 9 points), inability to comply with the study protocol or to provide written informed consent. Further exclusion criteria were chronic bedridden conditions, physical handicap, severe neuromuscular disease, and use of drugs affecting body composition (such as glucocorticoids, statins, active vitamin D metabolites, anabolic steroids, selective estrogen receptor modulators). The study was approved by our Institutional Review Board at the Ospedali Riuniti in Foggia and performed according to the Declaration of Helsinki. All patients gave written informed consent.

### 2.2. Biochemical Analysis, Anthropometric Measurements and Body Composition Evaluation

A blood sample was taken at the time of admission for the determination of hemoglobin (Hb) and lymphocytes in whole blood, and total proteins, albumin, total cholesterol, and iron in the serum. Height, body weight, and waist, arm, and hip circumference, as well as tricipital, bicipital, subscapular, and supra-iliac skinfold thicknesses were measured according to standardized procedures. Body mass index (BMI) was calculated as the ratio between weight in kilograms and the square of height in meters. Body composition was assessed within 24 h from admission by bioelectrical impedance using a BIA 101-F device (Akern/RJL, Florence, Italy), as previously reported [17]. The BIA analyzer underwent calibration by the manufacturer, and the measurements were validated according to previously published equations [18].

### 2.3. Tools for Screening of Nutritional Status

The MUST, SGA, and NRS-2002 tools were used for nutritional screening, and the time (in seconds) required to complete each test was recorded. To avoid any interindividual variance, all tools were performed by an experienced operator.

The MUST includes three clinical parameters and rates each parameter as 0, 1 or 2 as follows: (a) BMI > 20 kg/m^2^ = 0; 18.5–20.0 kg/m^2^ = 1; <18.5 kg/m^2^ = 2; (b) weight loss in the past 3–6 months < 5% = 0; 5–10% = 1; >10% = 2; (c) acute disease: absent = 0; if present = 2. Overall risk of malnutrition is established as follows: 0 = low risk; 1 = medium risk; 2 = high risk [19].

The SGA questionnaire includes patient history (weight loss, changes in dietary intake, gastrointestinal symptoms and functional capacity), physical examination (muscle, subcutaneous fat, sacral and ankle edema, ascites) and the clinician’s overall judgment of the patient status ((a) well nourished; (b) suspected malnourished or moderately malnourished; (c) severely malnourished) [20].

The NRS-2002 consists of a nutritional score and a severity of disease score and an age adjustment for patients aged > 70 years (+1). Nutritional score: weight loss 45% in 3 months or food intake below 50–75% in the preceding week = 1; weight loss 45% in 2 months or BMI 18.5–20.5 kg/m^2^ and impaired general condition or food intake 25–60% in the preceding week = 2; weight loss 45% in 1 months or >15% in 3 months or BMI < 18.5 kg/m^2^ and impaired general condition or food intake 0–25% in the preceding week = 3. Severity of disease score: hip fracture, chronic patients with acute complications = 1; major abdominal surgery, stroke, severe pneumonia, hematological malignancies = 2; head injury, bone marrow transplantation, intensive care patients with APACHE > 10 = 3. NRS-2002 score is the total of the nutritional score, severity of disease score and age adjustment. Patients are classified at no risk = 0, low risk = 0–1, medium risk = 3–4, and high risk = ≥ 5 [21].

### 2.4. Diagnostic Criteria for Malnutrition and Sarcopenia

Following the new GLIM diagnostic criteria, malnutrition was diagnosed when the patients met 1 phenotypic criterion (among non-volitional weight loss, low body mass index, and reduced muscle mass) and 1 etiologic criterion (among reduced food intake or assimilation, and disease burden/inflammatory condition), according to Supplementary Table S2 [8].

Sarcopenia was diagnosed on admission according to the European Working Group on Sarcopenia in Older People updated recommendation (EWGSOP2) [22]. Particularly, sarcopenia was first assessed by gait speed or grip strength, and then, confirmed in subjects presenting with a relative skeletal muscle index (RSMI) < 7.25 kg/m^2^ (men) or <5.67 kg/m^2^ (women).

### 2.5. Statistical Analysis

Data were expressed as mean ± standard deviation of the mean (SDM) for quantitative variables, and as count and percentages for qualitative values. Gaussian distribution of the samples was evaluated by the Kolgomorov–Smirnov test. The significance of differences between 2 groups was assessed by Student’s *t*-test (continuous variables) or in contingency tables by Pearson’s Chi-squared test and Fisher’s exact test (categorical variables). The significance of differences between more than 2 groups was assessed by the one-way analysis of variance (ANOVA) after ascertaining normality by the Kolgomorov–Smirnov test; Tukey–Kramer was applied as post hoc test. To determine the diagnostic concordance between the three screening tools and the GLIM diagnostic criteria for malnutrition, Cohen’s к statistic was calculated. The к coefficient reflects the consistency of qualitative variables: к = 1 indicates complete consistency between the variables, and к = 0 indicates no consistency among the variables. Positive likelihood ratios and negative likelihood ratios were calculated for all three tools. Sensitivity and specificity values for the three nutritional screening tools with the GLIM diagnostic criteria for malnutrition were calculated. To determine the diagnostic concordance, consistency, accuracy, likelihood ratio, sensitivity, and specificity, medium and high-risk categories for the three nutritional assessment tools were combined, according to previous publications [23,24]. Receiver operating characteristic (ROC) curves of the three screening tools were also used to evaluate the ability to accurately distinguish malnourished patients. The Youden Index was calculated as (sensitivity + specificity) − 1 for each cut-off point. The odds ratio (OR) and the 95% confidence interval (CI) were calculated. Univariate binary logistic regression analysis was used to analyze the association between nutritional status and the presence of sarcopenia.

All tests were 2-sided, and *p* values <0.05 were considered statistically significant. Statistical analysis was performed with the Statistical Package for Social Sciences version 23.0 (SPSS, Inc., Chicago, IL, USA) and the package GraphPad Prism 6.0 for Windows (GraphPad Software, Inc., San Diego, CA, USA).

## 3. Results

### 3.1. Patients Characteristics

**Figure 1:**
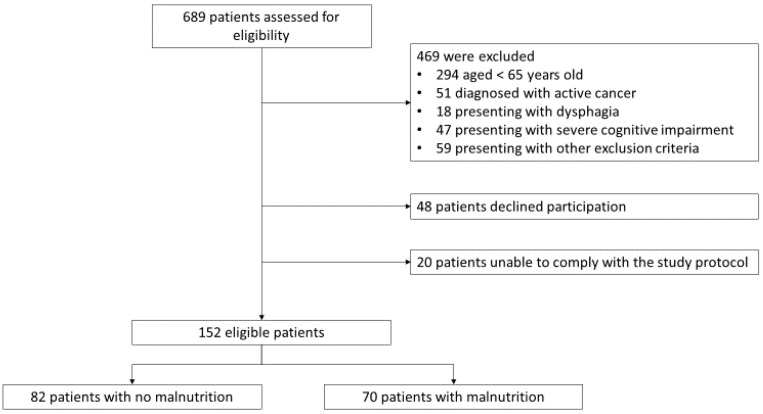
Participant flowchart. Other exclusion criteria were chronic bedridden conditions, physical handicap, severe neuromuscular disease, and use of drugs affecting body composition (such as glucocorticoids, statins, active vitamin D metabolites, anabolic steroids, selective estrogen receptor modulators). Diagnosis of malnutrition was performed according to the new criteria of the Global Leadership Initiative on Malnutrition.

In total, 689 consecutive patients were evaluated for enrolment; of these, 152 met the inclusion criteria and did not present any of the exclusion criteria. According to the GLIM criteria, malnutrition was diagnosed in 70 patients (46%) at admission (Figure 1, Supplementary Table S3).

Baseline demographic, clinical, anthropometric, and biochemical characteristics of patients presenting with malnutrition or not malnourished are represented in Table 1. Of note, we reported lower weight, arm and waist circumference, waist-to-hip ratio, bicipital, subscapular and supra-iliac skinfold thickness, serum total proteins, serum albumin, serum total cholesterol and blood hemoglobin in the group of patients with malnutrition with respect to the group with no malnutrition.

Interestingly, significant differences in body composition parameters were observed between the two groups (Table 2). In detail, patients with malnutrition were observed with reduced Body Cell Mass, Fat-Free Mass, Skeletal Muscle Index and Appendicular Skeletal Muscle Mass, and increased Total Body Water, Extracellular Water and Fat Mass as compared with not malnourished patients.

### 3.2. Rapidity, Sensitivity, Specificity, Accuracy, and Diagnostic Value of Nutritional Screening Tools

**Figure 2:**
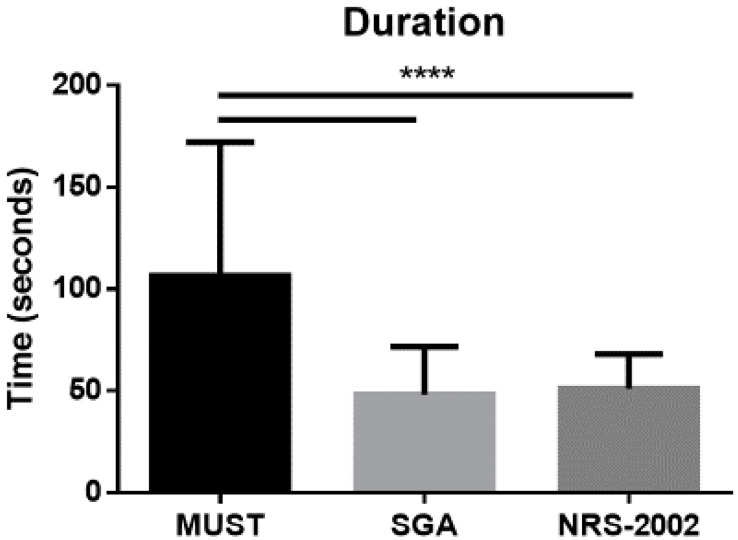
Duration of the nutritional screening tools (time expressed in seconds), performed in all the 152 hospitalized patients enrolled in this study. Data are expressed as mean ± SD. Statistical differences were assessed by one-way ANOVA and Tukey as post hoc test. MUST, Malnutrition Universal Screening Tool; SGA, Subjective Global Assessment; NRS-2002, Nutritional Risk Screening 2002. ****: *p* < 0.0001 vs. MUST.

All patients were subjected to three nutritional screening tools (MUST, SGA, and NRS-2002) at admission. As shown in Figure 2, MUST was the less rapid tool as compared to SGA and NRS-2002.

The MUST misclassified 18%, the SGA 47%, and the NRS-2002 38% of patients. Sensitivity was 64.3% with the MUST, 95.7% with the SGA, and 47.1% with the NRS-2002, while specificity was 81.7%, 14.6% and 75.6% with the MUST, SGA and NRS-2002, respectively; MUST accuracy was 73.7%, while accuracy resulted 52% for SGA and 62.5% for NRS-2002 (Table 3).

**Figure 3:**
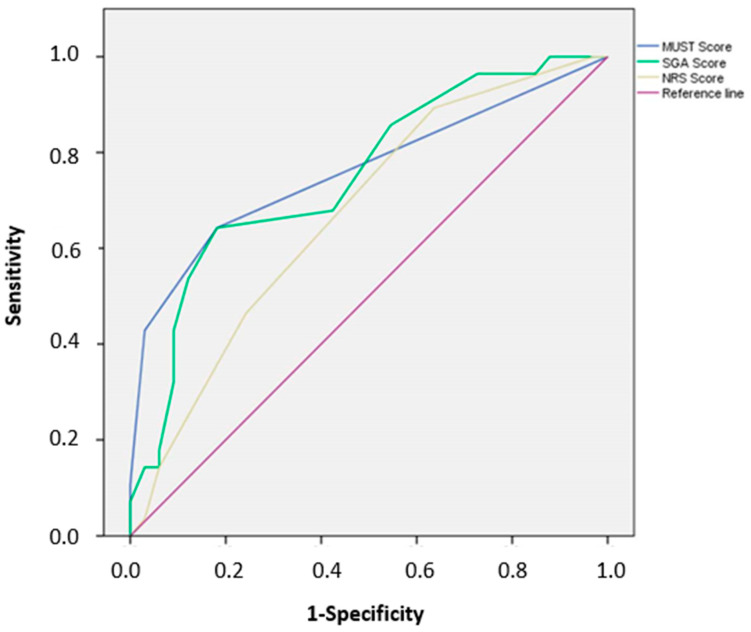
Receiver Operating Characteristic (ROC) curve for prediction of malnutrition based on the score obtained by the Malnutrition Universal Screening Tool (MUST, blue line), the Subjective Global Assessment (SGA, green line), and the Nutritional Risk Screening 2002 (NRS-2002, yellow line).

Finally, the area under the curve (AUC) calculated by the ROC indicated that all three screening tools had a moderate value to diagnose malnutrition in hospitalized older patient (AUC of MUST, SGA, and NRS-2002 were found to be 0.80, 0.77 and 0.69, respectively; Figure 3). The highest Youden indexes were 0.461 for a MUST score ≥ 0.5 (sensitivity 0.643, specificity 0.818), 0.461 for a SGA score ≥ 8.5 (sensitivity 0.643, specificity 0.818), and 0.257 for a NRS-2002 score ≥ 2.5 (sensitivity 0.464, specificity 0.758).

### 3.3. Malnutrition and Sarcopenia

According to the EWGSOP2 recommendation, sarcopenia was diagnosed in 77 (50.6%) patients; of these, 45 (64.3%) were also diagnosed with malnutrition according to the GLIM criteria, and 32 (39.0%) were not malnourished (Χ_2_ = 9.641, *p* = 0.0019). We did not find any difference between genders with respect to the diagnosis of sarcopenia. Malnutrition diagnosed according to the GLIM criteria increased the risk of presenting with sarcopenia 2.7-fold (95% CI 1.4–4.9, *p* = 0.0029). There was a significant association between sarcopenia and nutritional status at high risk of malnutrition detected by MUST, but not by other nutritional screening tools (Table 4).

## 4. Discussion

This is the first study which compared the diagnostic reliability of different nutritional screening tools to the GLIM criteria in a population of hospitalized older patients. According to the GLIM framework, 46% of patients were identified as malnourished when using combinations of two criteria. This prevalence could be apparently high as compared with previous studies which used the GLIM criteria for the diagnosis of malnutrition [5,25,26,27]. Nevertheless, this investigation focused on a special population that presented with a high prevalence of etiologic GLIM criteria for malnutrition. Indeed, the prevalence of malnutrition is close to 50% when diagnosed in critically ill patients [28]. Furthermore, even though this study was not designed to validate the diagnosis of malnutrition in hospitalized older subjects according to the GLIM consensus, our data show that patients with malnutrition presented with alterations in anthropometry, biochemical parameters, and bioelectrical impedance analysis results, compatible with their impaired nutritional status.

Nutritional screening tools detect features associated with alterations of nutritional status to distinguish persons presenting with risk of malnutrition. These tools play an important role in providing a standardized and systematic approach to identifying malnutrition [29]. Considering the utility of such tools in a daily routine, they should be easy to use, rapid, sensitive, and specific, to be included in a defined clinical protocol [30]. In this study, we compared the MUST, created to identify malnutrition in care settings [31]; the SGA, considered by several Authors as the most validated tool in hospital settings [25]; the NRS-2002, a tool used in hospital settings to detect patients who would benefit from nutritional therapy [21]. We first considered the rapidity of each tool in our special population, concluding that the MUST requires longer time with respect to both SGA and NRS-2002. Medium and high nutritional risk was 39.5% by MUST, 90.1% by SGA, and 34.9% by NRS-2002, compared to 46% of patients being malnourished by GLIM consensus. Concordance of the three screening tools with respect to GLIM criteria resulted dissimilar. These data are similar to other comparisons between different nutritional screening tools applied to hospitalized older patients [32]. Overall, the three screening tools misclassified 11–47% of patients, the MUST having the highest concordance with GLIM framework compared with SGA and NRS-2002. Furthermore, the MUST showed higher specificity than SGA and NRS-2002, meaning that more hospitalized older patients presenting with no malnutrition were correctly identified as at a low risk of malnutrition than malnourished patients being at high risk. On the contrary, the SGA showed higher sensitivity with respect to MUST and NRS-2002, properly identifying hospitalized older patients with malnutrition as at high risk than well-nourished ones at low risk. A previous study analyzed the validity of GLIM criteria with respect to SGA, describing a higher sensitivity when weight loss was combined with high C-reactive protein values, and a higher specificity with the combination of a low BMI and low food intake [25]. Both reduced food intake and increased C-reactive protein were registered very frequently in our population of hospitalized older patients, however, weight loss was more prevalent than low BMI, partially explaining the high sensitivity (but low specificity) of SGA. Finally, even though the ROC curve showed a moderate value to identify malnutrition by all three nutritional screening tools, MUST was found to have the greatest AUC with respect to SGA and NRS-2002. Based on the results of this study, the time spared in the administration of SGA or NRS-2002 is not well matched with accuracy or diagnostic value of these tools in hospitalized older patients. Further investigations are needed to confirm this observation.

This study reported a significant prevalence of sarcopenia diagnosed with the EWGSOP2 criteria, with malnutrition diagnosed with the GLIM criteria (64.3%). Malnutrition is considered as a determinant factor for the onset of sarcopenia, and the presence of malnutrition-sarcopenia syndrome (MSS) is associated with a four times higher risk of mortality in hospitalized older patients [6]. A recent longitudinal study investigated the incidence of sarcopenia (identified through the EWGSOP2 criteria) during a four-year follow-up in community-dwelling older individuals diagnosed with malnutrition at baseline according to both the European Society of Clinical Nutrition and Metabolism (ESPEN) and the GLIM criteria [5]. This report registered a threefold higher risk of developing sarcopenia in malnourished patients based on the GLIM [5]. Even though the present investigation is not designed as a prospective longitudinal study, our analysis shows that the risk of MSS is almost threefold in hospitalized older patients presenting with malnutrition at admission. Moreover, amongst the three screening tools applied, MUST is the only to identify older hospitalized patients at high risk of malnutrition with a significant 2.5 times higher risk of having MSS.

The strengths of this study consist in the use of standardized diagnostic criteria for the diagnosis of malnutrition or sarcopenia, and a rigorous statistical analysis. Nevertheless, this study presents several main limitations. First, it is a single center study with a small sample size, which restricted the subgroup analysis. Moreover, this study did not investigate the association between nutritional status and the underlying disease that led to hospitalization. The three nutritional screening tools were not compared with MNA, because of biased questions inappropriate to hospitalized older people even with mild or moderate cognitive impairment, and longer time to be performed. Muscle mass was evaluated by bioelectrical impedance, which could be influenced by fluid distribution changes. A further limitation of the study is that information about the dietary regimens or the pharmacological treatments potentially affecting the nutritional status of single participants was not registered. Moreover, in our study, we did not estimate other confounding factors, such as socioeconomic and family status, which could partially impact the results.

In conclusion, the present study reports a prevalence of malnutrition of 46% in hospitalized older patients, according to the GLIM framework criteria. The comparison of three different nutritional screening tools indicates that MUST is better at detecting malnutrition in hospitalized older patients diagnosed by the new GLIM criteria despite being less rapid, with respect to SGA and NRS-2002. Furthermore, there is a significant association between the presence of sarcopenia in hospitalized older patients at high risk of malnutrition according to MUST. This evidence confirms the importance of routine nutritional assessment in hospitalized older patients. We suggest that nutritional status should be determined by MUST in older patients at hospital admission, followed by both GLIM and EWGSOP2 assessment. The choice of this diagnostic tool could allow on-time nutritional intervention, thus preventing the worsening of negative caloric balance and loss of muscle mass. Furthermore, an early and valuable recognition of malnutrition and sarcopenia would be beneficial to plan individualized treatment during hospitalization and at discharge.

## Figures and Tables

**Table 1:** Baseline demographic, clinical, anthropometric, and biochemical characteristics of patients, stratified according to the Global Leadership Initiative on Malnutrition consensus.

Characteristic	No Malnutrition *n* = 82 (54%)	Malnutrition *n* = 70 (46%)	*p*
Age (years)	77.8 ± 7.8	78.7 ± 7.3	0.4664
Genre M/F (*n*, %)	47/35 (57/43)	40/30 (57/43)	0.9827
Education (*n*, %)			
None	0 (0)	0 (0)	0.8812
Primary School	45 (55)	40 (57)	
Secondary School	17 (21)	17 (24)	
High School	17 (21)	10 (14)	
University	3 (3)	3 (5)	
Co-morbidities > 3 (*n*, %)	37 (45)	32 (46)	0.9417
MMSE score	21.4 ± 6.6	19.9 ± 5.8	0.1420
Weight (kg)	76.7 ± 14.2	71.8 ± 16.0	**0.0473**
Height (m)	1.63 ± 0.1	1.62 ± 0.08	0.5021
BMI (kg/m^2^)	28.9 ± 5.9	27.4 ± 5.5	0.1091
Arm circumference (cm)	28.6 ± 4.0	27.2 ± 4.4	**0.0417**
Waist circumference (cm)	104.2 ± 10.7	99.7 ± 13.0	**0.0206**
Hip circumference (cm)	100.6 ± 10.5	99.2 ± 11.6	0.4159
Waist-to-Hip ratio	1.04 ± 0.11	1.00 ± 0.08	**0.0126**
Tricipital skinfold thickness (cm)	13.2 ± 4.7	12.6 ± 5.4	0.4651
Bicipital skinfold thickness (cm)	11.5 ± 4.6	9.8 ± 5.7	**0.0437**
Subscapular skinfold thickness (cm)	16.5 ± 6.2	14.0 ± 5.2	**0.0085**
Supra-iliac skinfold thickness (cm)	18.4 ± 7.0	13.7 ± 5.2	**<0.0001**
Total proteins (g/dL)	6.4 ± 0.6	6.0 ± 0.8	**0.0006**
Albumin (g/dL)	3.3 ± 0.5	3.1 ± 0.6	**0.0265**
Total Cholesterol (mg/dL)	147 ± 36	116 ± 52	**<0.0001**
Lymphocytes (n × 10^3^/mm^3^)	1.9 ± 2.5	2.6 ± 3.9	0.1837
Hemoglobin (g/dL)	11.8 ± 2.9	9.6 ± 1.9	**<0.0001**
Iron (mg/dL)	53.4 ± 32.7	47.3 ± 36.0	0.0839

Data are expressed as mean ± SD (continuous variables) or frequency and percentage (categorical variables). Statistical differences were assessed by Student’s *t*-test (continuous variables) or by Pearson’s Chi-squared test and Fisher’s exact test (categorical variables). M, male; F, female; MMSE, Mini-Mental State Examination. Bold: statistically significant.

**Table 2:** Bioelectrical impedance analysis parameters in patients stratified according to the Global Leadership Initiative on Malnutrition consensus.

Parameter	No Malnutrition *n* = 82 (54%)	Malnutrition *n* = 70 (46%)	*p*
Body Cell Mass (kg)	18.8 ± 6.6	10.4 ± 9.2	**<0.0001**
Total Body Water (L)	37.5 ± 5.5	41.2 ± 9.4	**0.003**
Extracellular water (L)	22.4 ± 3.9	28.9 ± 10.1	**<0.0001**
Fat-Free Mass (%)	63.2 ± 9.4	58.2 ± 11.4	**0.0035**
Fat Mass (%)	36.8 ± 9.4	41.8 ± 11.4	**0.0035**
Skeletal Muscle Index	8.2 ± 1.3	7.3 ± 1.9	**0.0007**
Appendicular Skeletal Muscle Mass (kg)	19.4 ± 3.7	16.5 ± 4.4	**<0.0001**
Appendicular Skeletal Muscle Mass (kg/m^2^)	7.32 ± 2.6	6.30 ± 2.0	**0.0083**

Data are expressed as mean ± SD. Statistical differences were assessed by Student’s *t*-test. Bold: statistically significant.

**Table 3:** Statistical comparison of nutritional diagnosis and screening tools values at hospital admission.

	MUST			SGA			NRS-2002		
GLIM	**Low Risk**	**Medium/High Risk**	**Total**	**Well Nourished**	**Moderately/Severely Malnourished**	**Total**	**Low Risk**	**Medium/High Risk**	**Total**
Well nourished	67	15	82	12	70	82	62	20	82
Malnutrition	25	45	70	3	67	70	37	33	70
Total	92	60	152	15	137	152	99	53	152
	**%**	**95% CI**		**%**	**95% CI**		**%**	**95% CI**	
Sensitivity	64.3	51.9–75.4		95.7	88.0–99.1		47.1	35.1–59.4	
Specificity	81.7	71.6–89.3		14.6	7.8–24.1		75.6	64.9–84.4	
Positive predictive value	75.0	62.1–85.3		48.9	40.3–57.6		62.3	47.9–75.2	
Negative predictive value	72.8	62.5 81.6		80.0	51.9–95.7		62.6	52.3–72.1	
Accuracy	73.7	66.7–80.7		52.0	44.1–59.9		62.5	54.8–70.2	
к	0.89			0.53			0.62		

Malnutrition Universal Screening Tool (MUST), Subjective Global Assessment (SGA) and Nutritional Risk Screening 2002 (NRS-2002) versus Global Leadership Initiative on Malnutrition (GLIM) criteria. CI, confidence interval; к statistic, percent of agreement.

**Table 4:** Association between nutritional status and presence of sarcopenia on admission.

Tool	Outcome	No Sarcopenia *n* (%)	Sarcopenia *n* (%)	OR (95% CI)	*p*
GLIM	No malnutrition	50 (61.0)	32 (39.0)	1	
	Malnutrition	25 (35.7)	45 (64.3)	2.7 (1.4–4.9)	**0.0029**
MUST	Low risk	60 (65.2)	32 (34.8)	1	
	Medium risk	8 (34.8)	15 (65.2)	0.6 (0.3–1.2)	0.459
	High risk	7 (18.9)	30 (81.1)	2.5 (1.3–3.6)	**0.0068**
SGA	Well nourished	9 (60.0)	6 (40.0)	1	
	Moderately malnourished	44 (51.8)	41 (48.2)	1.4 (0.3–4.1)	0.516
	Severely malnourished	22 (42.3)	30 (57.7)	2.7 (0.2–9.4)	0.414
NRS-2002	Low risk	53 (53.5)	46 (46.5)	1	
	Medium risk	12 (57.1)	9 (42.9)	0.1 (0.0–1.1)	0.059
	High risk	16 (47.0)	18 (53.0)	1.2 (0.2–5.8)	0.835

Incidence and odds ratio (OR; 95% confidence interval, CI), adjusted for age, gender, and education, between Global Leadership Initiative on Malnutrition (GLIM) diagnosis of malnutrition or nutritional screening tools and presence of sarcopenia. MUST, Malnutrition Universal Screening Tool; SGA, Subjective Global Assessment; NRS-2002, Nutritional Risk Screening 2002. Bold: statistically significant.

## Supplementary Materials

The following are available online at https://www.mdpi.com/2077-0383/9/6/1898/s1, Table S1: Main causes for hospital admission in patients stratified according to the Global Leadership Initiative on Malnutrition consensus. Table S2: Assessment criteria of malnutrition according to the Global Leader Initiative on Malnutrition (GLIM). Table S3: Prevalence of criteria of the Global Leader Initiative on Malnutrition (GLIM) registered in the study population.
