# Supplementary material for: Comparison of Three Nutritional Screening Tools with the New Glim Criteria for Malnutrition and Association with Sarcopenia in Hospitalized Older Patients
Source: J Clin Med. 2020 Jun 17;9(6):1898. doi: 10.3390/jcm9061898 (PMC7356988; doi:10.3390/jcm9061898)
Supplement: Supplementary file 1 [file jcm-09-01898-s001.pdf]

Table S1. Main causes for hospital admission in patients stratified according to the Global Leadership Initiative on Malnutrition consensus.

	**No malnutrition** *n* **= 82**	**Malnutrition** *n* **= 70**	***p***
**Heart failure (*n*, %)**	34 (41)	30 (43)	0.8707
**Respiratory failure (*n*, %)**	12 (15)	9 (13)	0.8167
**Anemia (*n*, %)**	7 (9)	11 (16)	0.2114
**Acute renal failure (*n*, %)**	7 (9)	3 (4)	0.3432
**Pneumonia (*n*, %)**	14 (17)	10 (14)	0.6631
**Acute pancreatitis (*n*, %)**	2 (2)	0 (0)	0.4998
**Other (*n*, %)**	6 (7)	7 (10)	0.5754

Statistical differences were assessed by Pearson’s Chi-squared test and Fisher’s exact test.

Table S2. Assessment criteria of malnutrition according to the Global Leader Initiative on Malnutrition (GLIM)

Phenotypic	Etiologic
Non-volitional weight loss<br>A weight loss > 4.5 kg in the past year was reported and used as a threshold. Unintentional weight loss was obtained by clinical interview at baseline.	*Reduced food intake or assimilation*<br>The Mini-nutritional Assessment- Short Form (MNA-SF) item was used to determine reduced food intake
Low body mass index<br>BMI (kg/m^2) was considered reduced if <20 kg/m^2 or <22 kg/m^2 in participants younger and older than 70 years, respectively.	*Disease burden/inflammatory condition*<br>Erythrocyte Sedimentation Rate and C-Reactive Protein were selected as biomarkers to assess inflammation
Reduced muscle mass<br>FFMI <17 kg/m^2 in men and <15 kg/m^2 in women or ALMI <7 kg/m^2 in men and <5.5 kg/m^2 in women was used as a threshold	

Diagnosis of malnutrition was performed when patients met at least 1 phenotypic and 1 etiologic criterion. BMI, Body Mass Index; FFMI, Fat-Free Mass Index; ALMI, Appendicular Lean Mass Index.

Table S3. Prevalence of criteria of the Global Leader Initiative on Malnutrition (GLIM) registered in the study population

**Phenotypic (*n* = 152)**	
1. *Non-volitional weight loss (n, %)*	48 (31.6)
2. *Low body mass index (n, %)*	27 (17.8)
3. *Reduced muscle mass (n, %)*	42 (27.6%)
**Etiologic (*n* = 152)**	
a. Reduced food intake or assimilation (n, %)	107 (70.4)
b. Disease burden/inflammatory condition (n, %)	96 (63.1)
**Combined criteria (*n* = 70)**	
• *1 + a (n, %)*	45 (64.3)
• *1 + b (n, %)*	43 (61.4)
• *2 + a (n, %)*	27 (38.6)
• *2 + b (n, %)*	25 (35.7)
• *3 + a (n, %)*	40 (57.1)
• *3 + b (n, %)*	39 (55.7)

The prevalence of single criteria is indicated for the whole study population, while the prevalence of combined criteria is related to patients diagnosed with malnutrition.
